# Coniferyl Aldehyde Inhibits the Inflammatory Effects of Leptomeningeal Cells by Suppressing the JAK2 Signaling

**DOI:** 10.1155/2020/4616308

**Published:** 2020-09-14

**Authors:** Yao Wang, Yajun Gao, Xue Li, Xiaolin Sun, Zhanqi Wang, Hanchi Wang, Ran Nie, Weixian Yu, Yanmin Zhou

**Affiliations:** ^1^Department of Oral Implantology, School and Hospital of Stomatology, Jilin University, Changchun 130021, China; ^2^VIP Integrated Department, School and Hospital of Stomatology, Jilin University, Changchun 130021, China; ^3^Jilin Provincial Key Laboratory of Tooth Development and Bone Remodeling, School and Hospital of Stomatology, Jilin University, Changchun 130021, China

## Abstract

**Background:**

The brain is in many ways an immunologically and pharmacologically privileged site because of the blood-brain barrier (BBB). But for chronic peripheral inflammation, inflammatory signals can be transmitted from the peripheral system into the central nervous system (CNS) through multiple channels and result in neuroinflammation. Leptomeningeal cells that form the BBB can trigger one signaling pathway by releasing cytokines to transmit inflammatory signals. Besides, the Janus kinase (JAK) family may have a certain function in the activation of leptomeninges. In the present study, we try to use coniferyl aldehyde (CA), a natural anti-inflammatory phenolic compound, to inhibit this inflammatory process and elucidate the underlying molecular mechanisms.

**Results:**

Secretion of proinflammatory cytokines (TNF-*α*, IL-1*β*, and IL-6) significantly increased after incubation with *P. gingivalis*. Moreover, TNF-*α*, IL-1*β*, and IL-6 levels were upregulated, and the JAK2 signaling was enhanced in leptomeningeal cells in a conditioned medium from activated macrophages, which leads to the immune response in microglia. However, this inflammatory effect of leptomeningeal cells was reversed by CA administration, accompanied by the decreased immune response in microglia. The western blot assay revealed that JAK2 phosphorylation was suppressed in leptomeningeal cells treated with CA.

**Conclusions:**

This study demonstrates that activated macrophages by *P. gingivalis* markedly induce the release of proinflammatory cytokines (TNF-*α*, IL-1*β*, and IL-6) from leptomeningeal cells, thereby activating the JAK2 signaling pathway and subsequently enhancing immune responses in microglia in the CNS. CA effectively inhibits the inflammatory effect of leptomeningeal cells via suppressing the JAK2 signaling pathway.

## 1. Introduction

Periodontitis (PD) is a chronic inflammatory disease caused by the presence of a bacterial biofilm called dental plaque, which has an impact on the periodontal ligaments and bone surrounding teeth [1]. It is estimated that severe PD afflicts ∼8% of the USA adults [2]. *P. gingivalis* is an anaerobic bacteria that inhabits the oral cavity and is considered to be the most common pathogenic bacterium in patients with chronic PD [3]. Evidences show that the polymicrobial synergy involving *P. gingivalis* in the oral cavity of PD patients causes host immune responses against *P. gingivalis* infections at systemic sites [4], followed by releases of inflammatory mediators into the blood circulation [5]. This indicates an underlying association between PD and neuroinflammation [5, 6]. However, there is still dispute over how inflammatory signals enter central nervous system (CNS) through blood-brain barrier (BBB).

Some researchers [7, 8] found that leptomeningeal cells which constitute the BBB may transduce inflammatory signals to inflammatory cells in the CNS in response to lipopolysaccharide (LPS) of *P. gingivalis*. The leptomeninges were always thought to be in the front line against infections to defend the CNS [9, 10], whereas meninges serve not only as a physical defense against infectious molecules, but also involve in signal transmission [11], molecule transport [12], stem cell incubation [11], and the immune responses in brain-resident inflammatory cells in the CNS [13]. Further evidence [14] shows that many substances, receptors, and cytokines, such as LPS, interleukin-1*β* (IL-1*β*), interleukin-6 (IL-6), cyclooxygenase (COX)-2, I*κ*B*α*, tumor necrosis factor *α* (TNF-*α*), and prostaglandin (PG), pronouncedly enhance secretion of more inflammatory factors from leptomeningeal cells. That means cytokines coming from leptomeningeal cells have been activated in the CNS even before the imminent entry of *P. gingivalis* into the brain. However, the exact mechanism has not been fully explored.

The immune response of CNS called neuroinflammation is characterized by the presence of activated microglia which has the unique capability to phagocytose toxic products, release cytotoxic factors, and behave as antigen-presenting cells [15–17]. Neuroinflammation is a common pathological process of multiple neurological diseases, such as neurodegenerative dementias [18, 19], intracerebral hemorrhage (ICH) [20, 21], and severe traumatic brain injury (sTBI) [22]. Chronic focal inflammation is considered to induce neuroinflammation through leptomeningeal cells. Epidemiological evidences show that early administration of nonsteroidal anti-inflammatory drugs (NSAIDs) reduces the risk of Alzheimer's disease (AD) [23].

As an important class of kinases in the human body, the Janus kinase (JAK) family is expressed in almost all cells. JAK2 as the most important phenotype and the most conserved isoform of the JAK family involves in various pathological processes. After activation of cell surface receptor induced by cytokines or LPS, JAK2 proteins will be recruited and phosphorylated, leading to the phosphorylation of the downstream STAT3. Phosphorylated STAT3 homodimerizes and translocates to the nucleus where it binds to the promoter of its target genes and activates their transcription to participate in fibrosis, angiogenesis, and inflammation in numerous diseases [24]. In our study, AG490 (a JAK2-specific inhibitor) was employed, aiming to figure out whether JAK2 involves in inflammation responses in leptomeningeal cells induced by cytokines from macrophages and whether it is a therapeutic target of neurodegenerative diseases.

Besides, we try to find out a new anti-inflammatory drug for the treatment of neuroinflammation and the resultant neurodegenerative diseases. Coniferyl aldehyde (CA) is the component of a natural non-toxic phenolic compound extracted from dietary and medicinal plants. It has always been used in the wine manufacture until recently its medical implication has been discovered [25–27].

The objectives of this study were to validate that leptomeningeal cells transmit inflammatory signals from blood-borne immune cells to brain-resident microglia and to expound the underlying mechanisms and drug intervention for the first time. We hypothesized that (1) *P. gingivalis* mediated the immune responses in macrophages; (2) secretions of macrophages elevated inflammatory levels of cytokines released from leptomeningeal cells; (3) the cytokines from leptomeningeal cells initiated inflammatory microglial responses; and (4) these inflammatory processes could be inhibited by CA via suppressing the JAK2/STAT signaling pathway.

## 2. Materials and Methods

### 2.1. Cell-Line Culture

The macrophage cell line RAW264.7 was purchased from the ATCC and the mouse microglia BV2 cell line from BeNa (Beijing, China). The cells were cultured in Dulbecco's Modified Eagle Medium (DMEM, Hyclone, USA) supplemented with 10% fetal bovine serum (FBS, Biological Industries, Israel Beit-Haemek), penicillin G, and streptomycin (100 *μ*g/mL, Hyclone, USA). The cells were incubated at 37°C in a humidified, 5% CO_2_ atmosphere.

### 2.2. Leptomeningeal Cell Culture

Preparations of leptomeningeal cell culture were described elsewhere [7, 28]. The brains of 3-day-old C57black/6N mice were collected and soaked in an ice-cold phosphate buffer saline (PBS). Leptomeningeal tissues were collected, plated on a poly-D-lysine-coated culture flask, and incubated in DMEM (Hyclone, USA) containing 10% FBS (Biological Industries, Israel Beit-Haemek), penicillin G, and streptomycin (100 *μ*g/mL, Hyclone, USA). After 7 days, the culture flask was strongly shaken and washed with a sterile, isotonic Ca^2+^/Mg^2+^-free buffer (pH 7.0), which consisted of 137 mM NaCl, 5 mM KCl, 0.7 mM KH_2_PO_4_, 25 mM glucose, 59 mM sucrose, 0.3% bovine serum albumin (BSA), and penicillin and streptomycin (100 *μ*g/mL). This step could remove any contaminated cells such as neuronal and glial cells [8, 29].

### 2.3. Macrophage Conditioned Medium (MCM) Was Collected to Analyze Cytokines by an ELISA

Macrophages were placed into a 10 cm culture dish (5 × 10^5^ cells/dish). The cells were treated with PBS, alive *P. gingivalis*, or *P. gingivalis* LPS (5 *μ*g/mL). Another set of cells was preincubated with AG490 (Sigma, USA), a JAK2-specific inhibitor (50 *μ*g/mL), for 12 h before treated with alive *P. gingivalis*. After 24 h of administration, supernatants of cultured macrophages passed through a filter to remove bacteria and cells. The levels of two classic cytokines in these supernatants were determined using an ELISA (R&D Systems, Inc., Minneapolis, MN, USA). The experimental procedure was performed in accordance to the manufacturer's instructions. The MCM in the *P. gingivalis* group was collected and stored at -20°C up to 2 weeks or -80°C up to 3 months. On the other hand, alive *P. gingivalis* was added to complete medium and stored at 37°C in a humidified, 5% CO_2_ atmosphere for 24 h. It was then filtered, and the supernatant was marked as “bacteria-free culture broth filtrate (BF)”. The two liquids above were both going to be used in the following experiment.

### 2.4. Leptomeningeal Cells Were Treated with MCM

Leptomeningeal cells were seeded in 6-well culture plates. The cells were incubated with complete medium, MCM, or BF that we had prepared in the previous experiment. Another set of cells was preincubated with AG490 before treated with MCM. The cells from all groups were incubated at 37°C for 6 h, and the medium was renewed. After another 6 h of incubation, the new medium was filtered, and the supernatant was collected. Cytokines in the leptomeningeal-cell-conditioned mediums (LCMs) of all groups were also analyzed by an ELISA, using the same procedures as previously described. Besides, mRNA expression levels of the three inflammatory cytokines in leptomeningeal cells were quantitated using real time quantitative polymerase chain reaction (RT-qPCR) analysis. Following the manufacturer's instructions, the total mRNA was extracted using a Hipure total mRNA Mini kit (Magen, China). A total of 1000 ng of extracted RNA was reversely transcribed to cDNA using the Primescript RT reagent Kit with gDNA Eraser (Takara, Japan). The thermal cycling was set at 42°C for 2 min, and then at 37°C for 15 min, followed by 85°C for 5 s. The cDNA was amplified in duplicate using the TB Green Premix Ex Taq II (Takara, Japan). The primer sequences used were listed in Table 1. Finally, all LCMs in MCM group, MCM+AG490 group, and BF group were collected and filtered. The supernatants were labeled as “LCM (MCM),” “LCM (MCM+AG490),” and “LCM (BF),” respectively, which were going to be used in the following experiment.

### 2.5. Microglia Were Treated with LCM

Microglia were treated with complete medium or LCM (MCM) or LCM (MCM+AG490) or LCM (BF) for 12 h. Then, the medium was renewed to remove the cytokine environment. The cells together with the new medium were collected after another 12 h of incubation. Protein and mRNA expression levels of inflammatory factors were determined using an ELISA and RT-qPCR, respectively.

### 2.6. Cytotoxicity of CA Was Evaluated Using the CCK-8 Assay and Fluorescent Staining

Leptomeningeal cells were plated into 96-well plates (1 × 10^4^ cells per well) and 6-well plates (1 × 10^4^ cells per well). They were incubated with 1, 5, and 50 *μ*M CA and PBS for 12 h. Leptomeningeal cells in 6-well plates were stained with FITC and DAPI to evaluate their morphological structure after CA administrations. The proliferation activity of the cell was determined using the cell-counting kit-8 (CCK-8) assay (Dojindo, Kumamoto, Japan). After 4 h of incubation with CCK-8, the OD value at 450 nm was measured by a microplate reader.

### 2.7. Inflammatory Responses of Leptomeningeal Cells after CA Administration Were Assessed Using RT-qPCR Analysis

Leptomeningeal cells pretreated with 1, 5, and 50 *μ*M CA for 12 h were cultured with MCM for another 12 h. Subsequently, the cells were collected, and IL-6 mRNA expression levels were determined by RT-qPCR analysis. The supernatants of all groups were collected and labeled as “LCM (MCM),” “LCM (MCM+1 *μ*M CA),” “LCM (MCM+5 *μ*M CA),” and “LCM (MCM+50 *μ*M CA),” and microglia were incubated with these conditioned mediums at 37°C for 24 h. The mRNA expression level of proinflammatory cytokine IL-6 was determined by RT-qPCR.

### 2.8. Phosphorylated JAK2 Levels in Leptomeningeal Cells after CA Administrations Were Analyzed Using Western Blot Assay

Leptomeningeal cells in the MCM+CA group were preincubated with 50 *μ*M CA for 12 h before the medium was replaced with MCM. The cells in the MCM group were only incubated with MCM. The cells in the control group were not treated with any special drug. The expressions of phosphorylated JAK2 in the three groups were quantitated using western blot analysis. The anti-p-JAK2 antibody was provided by Sigma (Merck KGaA, Darmstadt, Germany) and the protein extraction kit by ABclonal (Wuhan, China).

### 2.9. Statistical Analysis

The data are represented as the means ± SEM (*n* = 3, each); all assays were repeated three times with independent samples. The GraphPad Prism (GraphPad Software, San Diego, CA, USA) was used to perform statistical analyses. One-way ANOVA or Student's *t*-test was used for comparisons between groups. A value of *P* < 0.05 was considered to indicate statistical significance.

## 3. Results

### 3.1. The Effect of *P. gingivalis* on the Inflammatory Factors from Macrophages

**Figure 1:**
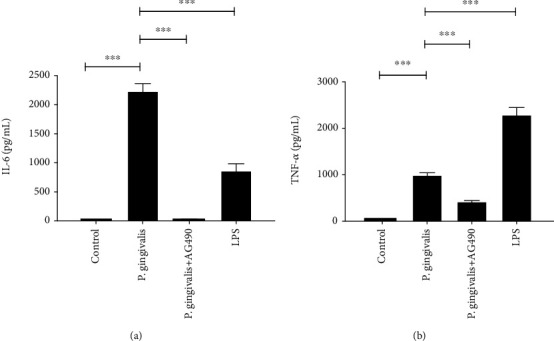
Inhibitory effects of AG490 on the secretion of proinflammatory cytokines by *P. gingivalis*-treated macrophages. (a, b) The mean concentrations of TNF-*α* and IL-6 in the culture medium of macrophages exposed to *P. gingivalis* or *P. gingivalis* LPS for 24 h were measured by ELISA. Each column and bar represent the mean ± SEM (*n* = 3, each). Asterisks indicate a statistically significantly difference from the value in corresponding group (∗∗∗*P* < 0.001).

To validate the immune responses in macrophages, we detected the inflammatory levels of cytokines in the medium. The protein levels of two cytokines TNF-*α* and IL-6 were significantly higher in the *P. gingivalis* group and the LPS group than those in the control group (*P* < 0.001) (Figure 1). Unexpectedly, these inflammatory cytokines showed distinct sensitivities to *P. gingivalis* and *P. gingivalis* LPS. The upregulation of TNF-*α* in macrophages treated with *P. gingivalis* was weaker than that in macrophages treated with *P. gingivalis* LPS. On the contrary, the upregulation of IL-6 was stronger in those treated with *P. gingivalis* than those in macrophages treated with *P. gingivalis* LPS. TNF-*α* and IL-6 levels significantly decreased in the *P. gingivalis*+AG490 group. These results confirmed that *P. gingivalis* stimulated inflammatory reactions of macrophages, and this effect was partially reversed when JAK2 was suppressed.

### 3.2. *P. gingivalis* Triggered Macrophage-Dependent Immune Response in Leptomeningeal Cells

**Figure 2:**
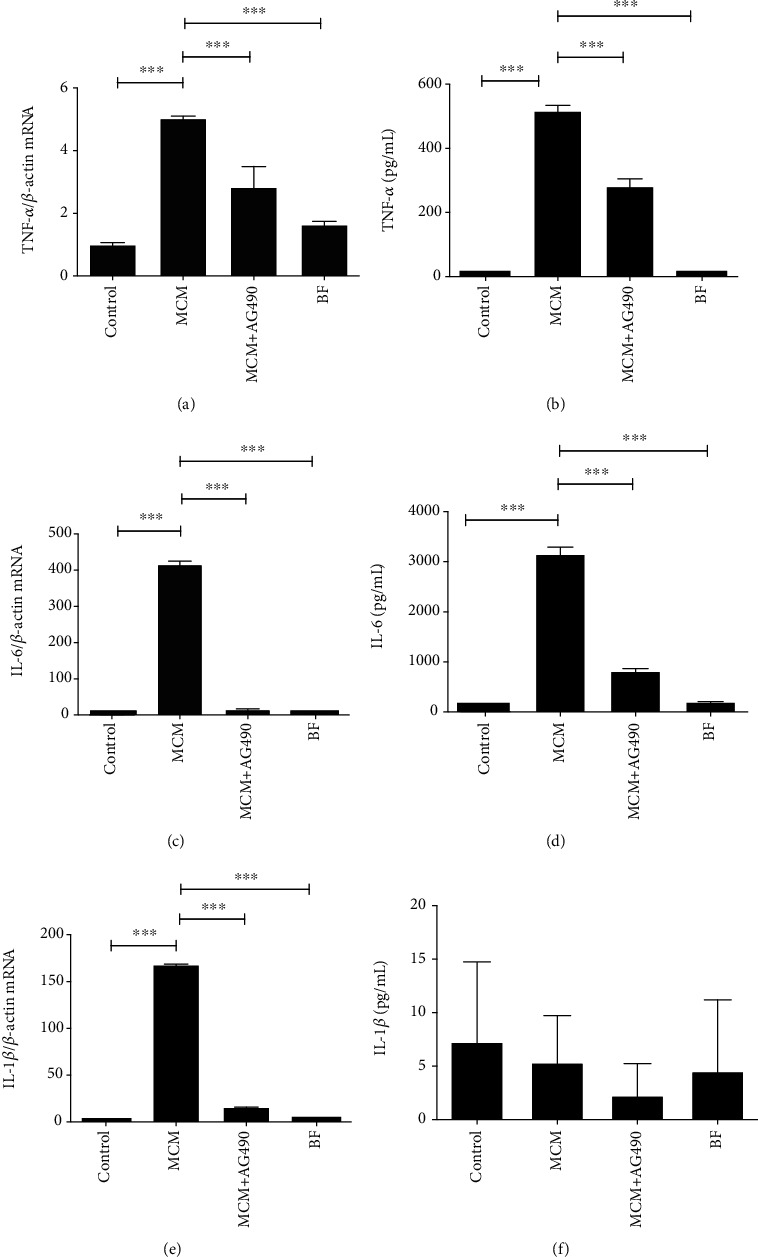
MCM increases the expression of proinflammatory mediators in leptomeningeal cells, which is suppressed by AG490. (a, b) TNF-*α* mRNA and protein levels in leptomeningeal cells after 12 h of treatment were measured by RT-qPCR and ELISA; (c, d) IL-6 mRNA and protein levels in leptomeningeal cells after 12 h of treatment were measured by RT-qPCR and ELISA; (e, f) IL-1*β* mRNA and protein levels in leptomeningeal cells after 12 h of treatment were measured by RT-qPCR and ELISA. Each column and bar represent the mean ± SEM (*n* = 3, each). Asterisks indicate a statistically significantly difference from the value in corresponding group (∗∗*P*<0.01; ∗∗∗*P* < 0.001).

After the inflammatory reaction of macrophages had been evaluated in the previous experiment, we tried to explore the influence of macrophages on leptomeningeal cells. TNF-*α*, IL-1*β*, and IL-6 mRNA expressions significantly increased in the MCM group compared to those in the control and negative control group (or the BF group) (Figure 2). Similar results were observed when we detected their protein levels using an ELISA. Surprisingly, protein levels of IL-1*β* were very small or even detected naught in some wells. There was no significant difference between all groups. For BF group, we found that its inflammatory stimulation was slight compared with the MCM group. This supported our hypothesis that macrophages rather than bacterial soluble metabolites triggered the inflammatory response in leptomeningeal cells.

On the other hand, AG490 (50 *μ*g/mL) was further used to understand the role of JAK2 in the inflammatory response of leptomeningeal cells. The levels of the three cytokines in the MCM+AG490 group were lower than those in the MCM group.

### 3.3. The LCM (MCM) Induced Microglial to Express Proinflammatory Cytokines

**Figure 3:**
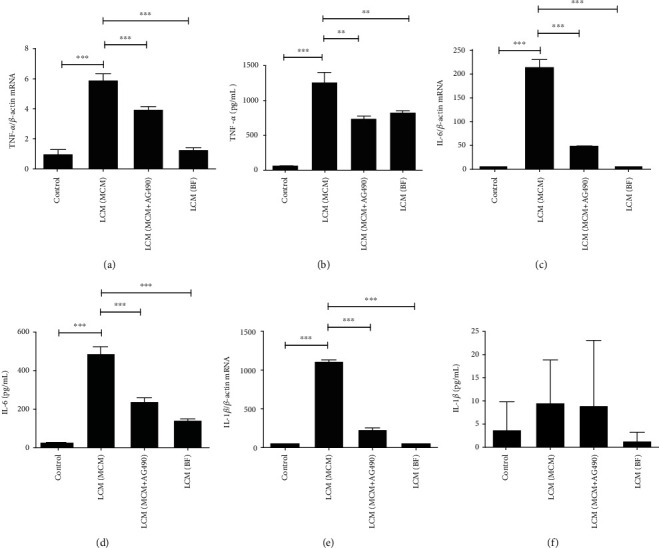
The effect of different LCMs on the expression of proinflammatory mediators in microglia. (a, b) TNF-*α* mRNA and protein levels in microglia after 24 h of incubation with LCMs or complete medium; (c, d) IL-6 mRNA and protein levels in microglia after 24 h of incubation with LCMs or complete medium; (e, f) IL-1*β* mRNA and protein levels in microglia after 24 h of incubation with LCMs or complete medium. Each column and bar represent the mean ± SEM (*n* = 3, each). Asterisks indicate a statistically significantly difference from the value in corresponding group (∗∗*P* < 0.01; ∗∗∗*P* < 0.001).

In order to understand the effect of activated leptomeningeal cells on immune microglial responses, the major immune cells in the brain, we used LCMs to stimulate the microglia and observe its reaction in this step. It was found that LCM (MCM) triggered immune responses in microglia. And the stimulating effects of LCM (MCM+AG490) and LCM (BF) on microglia were weaker than the effect of LCM (MCM) (Figure 3). This meant the activity of inflammatory signals was inhibited with the presence of AG490 (50 *μ*g/mL). Similarly, LCM (MCM) did not increase IL-1*β* protein levels in the medium even though its mRNA levels in the LCM (MCM) group were much higher than those in the control group.

### 3.4. Cytotoxicity of CA to Leptomeningeal Cells Was Negligible

**Figure 4:**
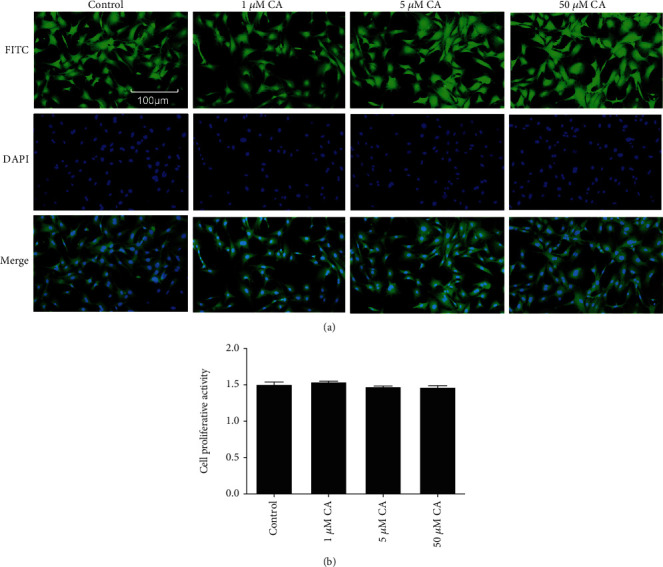
The effect of CA on morphology and proliferation of leptomeningeal cells was negligible. (a) Fluorescent staining under a microscope. Green represented cytoplasmic proteins stained with FITC, and blue represented the nuclei stained with DAPI. (b) CCK8 assay evaluated that the proliferation rates in leptomeningeal cells after 24 h of 1, 5, and 50 *μ*M CA treatment. Each column and bar represent the mean ± SEM (*n* = 3, each). There was no significant difference between groups.

In order to confirm the optimal dose of CA, which showed a potent anti-inflammation effect without affecting the cellular activity, the cytotoxicity of CA was assessed. After 24 h of 1, 5, 50 *μ*M CA treatment, immunofluorescence analysis showed that all cells were long-fusiform shapes or irregular polygons, and the nuclei were round or oval. There were no significant changes in the morphology of the cells between groups under a microscope (Figure 4(a)). There was a nonsignificant difference in cell proliferative between CA groups and the control group (Figure 4(b)). This suggested that 1, 5, and 50 *μ*M CA treatment had negligible effects on the morphology and proliferation of leptomeningeal cells.

### 3.5. CA Suppressed the Inflammatory Reaction of Leptomeningeal Cells

**Figure 5:**
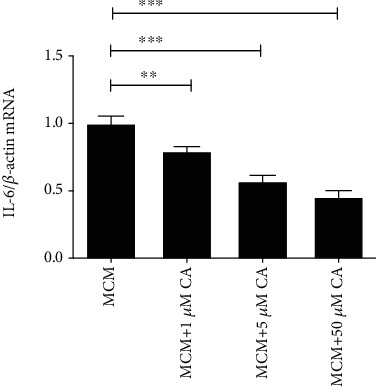
Inhibitory effects of AG490 on the expression of IL-6 mRNA in leptomeningeal cells treated with MCM. The IL-6 mRNA levels in leptomeningeal cells after 12 h of treatment were measured with RT-qPCR. Each column and bar represent the mean ± SEM (*n* = 3, each). Asterisks indicate a statistically significantly difference from the value in corresponding group (∗∗*P* < 0.01; ∗∗∗*P* < 0.001).

The leptomeningeal cells incubated with MCM+CA showed a lower IL-6 mRNA level than those treated with MCM. As the concentration of CA increased, IL-6 mRNA levels decreased (Figure 5).

**Figure 6:**
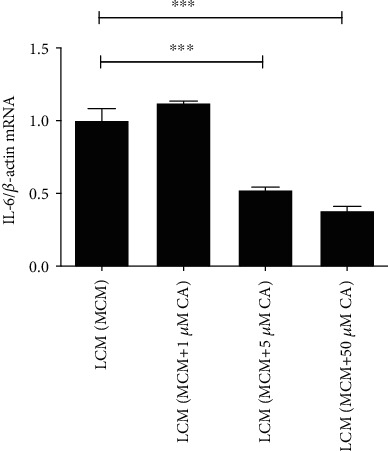
The effects of different LCMs on the expression of IL-6 mRNA in microglia. The IL-6 mRNA levels in microglia after 24 h of treatment were measured with RT-qPCR. Each column and bar represent the mean ± SEM (*n* = 3, each). Asterisks indicate a statistically significantly difference from the value in corresponding group (∗∗∗*P* < 0.001).

For microglia, there was a nonsignificant difference in the IL-6 mRNA level between the LCM (MCM) and LCM (MCM+1 *μ*M CA) groups. But IL-6 levels in the LCM (MCM+5 *μ*M CA) and LCM (MCM+50 *μ*M CA) groups were both lower than the level in the LCM (MCM) group (Figure 6).

### 3.6. CA Partially Inhibited p-JAK2 Expressions in Leptomeningeal Cells Incubated with MCM

**Figure 7:**
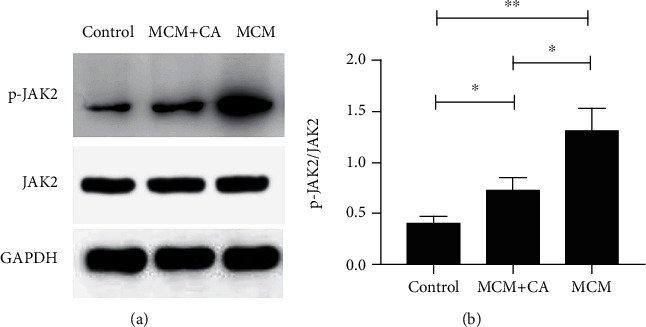
Inhibitory effect of CA on expressions of p-JAK2 protein in leptomeningeal cells activated by MCM. (a) The protein expressions of p-JAK2 and JAK2 in leptomeningeal cell were quantitated using western blot assays. (b) The quantitative analyses of the immunoblotting for p-JAK2/JAK2 in (a). Each column and bar represent the mean ± SEM (𝑛 = 3, each). Asterisks indicate a statistically significantly difference from the value in corresponding group (∗*P* < 0.05; ∗∗*P* < 0.01).

To identify the exact signaling pathway involving in the anti-inflammatory activities of CA, we assessed the phosphorylation level of JAK2 in the MCM+CA, MCM, and control groups using western blot assay. After 12 h of CA incubation, the p-JAK2/JAK2 level in the MCM+CA group was higher than that in the control group (Figure 7). However, the p-JAK2/JAK2 level significantly decreased in the MCM+CA group compared with the MCM group. There were significant differences between the groups.

## 4. Discussion

Although LPS and proinflammatory factors as large molecules cannot easily penetrate the BBB, many clinical studies [30–32] have pointed out a potential link between chronic PD, a common chronic systemic inflammation, and Alzheimer's disease (AD). Evidences suggest that the presence of systemic inflammation may predate *β*-amyloid deposition, which is considered as the cause of AD [33]. This indicates that therapy aiming at reducing inflammatory responses may contribute to mild cognitive impairment such as AD [34]. Though these efforts focusing on the need to ameliorate central inflammation have been disappointing, little attention has been paid to the importance of dampening down systemic inflammation. The further exploration about the communication between the periphery and the brain is clearly warranted. What we did in our experiment was to prove the ability of leptomeningeal cells to transmit the inflammatory signals coming from macrophages to microglia. The underlying mechanism and the possible therapies were also revealed in our *in vitro* experiment.

It is reported that activated macrophages are polarized to two major phenotypes [35]. M1 macrophages have proinflammatory function that increases expressions of TNF-*α*, Il-1*β*, IL-6, HLA-DR, and inducible nitric oxide synthase (iNOS), and M2 macrophages express transforming growth factor (TGF-*β*) and IL-10 to play an anti-inflammatory role [36–38]. *P. gingivalis* LPS promotes M1 macrophage polarization. But current researches indicate that gingipains, pili, and capsule all involve in chronic PD [31, 39]. *P. gingivalis* as anaerobic bacteria lives in the gingival crevicular fluid that directly contacts oxygen. Its effect on macrophages in oxygen-rich condition is complicated and rarely reported.

We treated mouse macrophages with *P. gingivalis* or LPS. As we expected, *P. gingivalis* stimulated the release of TNF-*α* and IL-6. This indicates that *P. gingivalis* activates mouse macrophages to express proinflammatory factors even in an aerobic environment. Furthermore, this stimulation is different from that induced by LPS. IL-6 is more sensitive to bacteria than LPS. But TNF-*α* is quite the opposite. That means actual proportions of cytokines expressed in human bodies can be different from the results from a laboratory.

Some researchers also showed that JAK2/STAT participates in immune responses in activated macrophages induced by *Staphylococcus aureus* and LPS [40, 41]. Our data also implied that the JAK2-specific inhibitor, AG490 (50 *μ*g/mL), downregulated expression levels of proinflammatory factors TNF-*α* and IL-6 in macrophages treated with *P. gingivalis* (Figure 1). This is the first report that shows a JAK2 inhibitor suppresses the proinflammatory effect of macrophages induced by *P. gingivalis*. It points out a new direction to reduce the level of systemic inflammation in patients with PD, but the exact mechanism is still not clear. It was reported that *P. gingivalis* induced the activation of the CatB/NF-*κ*B signaling in inflammatory macrophages [42]. Another research showed that LPS stimulated the expression of NF-*κ*B subunit p65 in human ciliary epithelial cells, resulting in the activation of receptor for growth-hormone-releasing hormone (GHRH-R). The downstream JAK2 is phosphorylated and augments the production of proinflammatory factors [43]. Therefore, we speculate that JAK2 is likely to be a downstream molecular target of CatB/NF-*κ*B in the macrophage-mediated inflammatory reaction induced by *P. gingivalis*. Obviously, this theory needs to be validated by more studies.

MCM resulted in inflammatory responses in leptomeningeal cells (Figure 2). It is worthy of mentioning that MCM has been filtered to get rid of alive bacteria and cells but not excluding soluble bacterial products. So, we set the BF group and noticed the enhanced secretion of TNF-*α* and IL-6 proteins, and mRNAs in the BF group were slightly stronger than that in the control group but significantly weaker than that in the MCM group. This indicates that there are the two components, proinflammatory factors from activated macrophages and soluble toxin from *P. gingivalis*, of MCM act on leptomeningeal cells, and proinflammatory factors have played a major role. In addition, the amount of IL-1*β* in the medium of all groups was so small that there was no significant difference between them (Figure 2(f)). Some bacterial metabolites can result in the ubiquitination and degradation of the inactive proform before mature IL-1*β* is produced [44]. This can be a probable explanation of this phenomenon. There is another possibility: except for primary stimulus like LPS, the secretion of IL-1*β* also depends on activators of the NLRP3 inflammasome, such as nigericin [45], and the activator can be deactivated in the aerobic medium. This speculation is also in need of verification.

Several inflammatory markers can be highly expressed in the serum of PD patients for a long time. Periodontal therapy did not seem to have any significant impact on the systemic cytokine levels [46]. That means leptomeningeal immune reaction will continue to persist in patients with chronic PD, even if they are asymptomatic in the face of cognitive impairment. A leptomeningeal inflammation can lead to structural changes, damages to the physical barrier, and the entry of immune cells into the CNS [47]. No doubt it is associated with further neuroinflammation and neurodegenerative disease.

Geng et al. [48] and Xiong et al. [49] showed that inactivated JAK2 reduced inflammatory responses in cardiac muscle cells and macrophages, which was conductive to finding out a new target therapy against cardiac hypertrophy and insulin resistance. For AD, JAK2 can induce neuroinflammation via the activation of glial cells [50, 51], but the function of JAK2 can be opposite in spinal cord injury [52] and chemical nerve injury [53]. Meanwhile, JAK2 is involved in local inflammation in PD [54]. Allowing for the intricate role of JAK2 in diseases of the nervous system, identifying the exact effect of JAK2 on leptomeninges in PD is of vital importance.

We found that the p-JAK2/JAK2 expressions in leptomeningeal cells significantly increased after MCM incubation (Figure 7). p-JAK2 induces phosphorylated STAT3 to homodimerize and translocate to the nucleus, which is associated to angiotensin II, interferon­*γ*, IL-6, transforming growth factor-*β* (TGF-*β*), iNOS, etc. [55, 56]. By binding to its receptor, IL-6 reactivates the JAK2 signaling in hepatic stellate cells (HSC) and upregulates itself, which forms a loop [57]. IL-6 was observed in MCM in our experiment (Figure 1), so we assumed that MCM could upregulate the proinflammatory factors of leptomeningeal cells by enhancing the JAK2 signaling. The obvious suppression of the inflammatory response induced by MCM was confirmed in leptomeningeal cells treated with AG490. This indicates that JAK2 is a novel target for therapy of inflammatory leptomeningeal reaction.

Furthermore, the medium coming from activated leptomeningeal cells triggered inflammatory activation of microglia, the key to neuroinflammation (Figure 3). This indicates that *P. gingivalis* in the peripheral system is able to cause CNS impairment and further neurodegenerative diseases. In a word, *P. gingivalis* induces macrophages to transmit inflammatory signals. Meanwhile, the JAK2-specific inhibitor blocks this process. These inflammatory signals subsequently activate leptomeningeal cells, initiating the immune response in microglia.

CA is a nontoxic compound extracted from natural plants. It is proved that CA can protect cells from radiation and neuroinflammation [58, 59]. Akram et al. [26] found the inhibitory effect of CA on paw edema of rat models induced by carrageenan (CRG). The effect on leptomeningeal cells has never been confirmed in the past reports. In our study, the CCK-8 assay and immunofluorescence analysis showed that the concentration of CA between 1 and 50 *μ*M did not affect the morphology or proliferation of leptomeningeal cells (Figure 4). So, it is proved that this concentration range is within a safe threshold to treat leptomeningeal cells. Immune responses in leptomeningeal cells induced by MCM were inhibited, and this inhibition was positively correlated with CA concentrations (Figure 5). This coincides with the decline in the inflammatory response in microglia activated by LCMs (Figure 6). This indicates a reduction in transmitting inflammation signals by CA, which has been confirmed *in vitro*. Besides, CA effectively suppressed the upregulations of p-JAK2/JAK2 in leptomeningeal cells treated with MCM (Figure 7). This suggests that the effect of CA on JAK2 phosphorylation is involved in this reduction. We will provide more precise verification in a timely manner in a subsequent publication.

## 5. Conclusions

*P. gingivalis* bacteria are able to induce the obvious inflammatory response in macrophages, and this induction can be reduced by JAK2 inhibitor. The cytokines coming from macrophages activate leptomeningeal cells, accompanied by upregulated the activities of JAK2 in leptomeningeal cells. Inflammatory activation of leptomeningeal cells can transfer immune signals to microglia cells and trigger a series of inflammatory responses in the CNS. CA can downregulate the levels of inflammatory cytokines in leptomeninges via suppressing JAK2 phosphorylation, which reduces the immune response in microglia and the CNS. That means CA may be the next generation of drugs for many neurodegenerative diseases.

## Figures and Tables

**Table 1:** Sequences of the primers used in RT-qPCR assays.

Target genes	Sequence
Actin-*β*	5′-CATCCGTAAAGACCTCTATGCCAAC-3′
	5′-ATGGAGCCACCGATCCACA-3′

TNF-*α*	5′-ACTCCAGGCGGTGCCTATGT-3′
	5′-GTGAGGGTCTGGGCCATAGAA-3′

IL-6	5′-CCACTTCACAAGTCGGAGGCTTA-3′
	5′-CCAGTTTGGTAGCATCCATCATTTC-3′

IL-1*β*	5′-TCCAGGATGAGGACATGAGCAC-3′
	5′-GAACGTCACACACCAGCAGGTTA-3′

## Data Availability

The figures and table data used to support the findings of this study are included within the article.
